# Ternary Interactions of Starch, Protein, and Polyphenols in Constructing Composite Thermoplastic Starch-Based Edible Packaging: Optimization of Preparation Techniques and Investigation of Film-Formation Mechanisms

**DOI:** 10.3390/foods15010036

**Published:** 2025-12-22

**Authors:** Anna Wang, Jingyuan Zhang, Ligen Wu

**Affiliations:** 1 School of Food Science and Technology, Henan University of Technology, Zhengzhou 450001, China; 2 National Engineering Research Center of Wheat and Corn Further Processing, Henan University of Technology, Zhengzhou 450001, China

**Keywords:** thermoplastic starch films, whey protein isolate, gallic acid, ternary interactions, edible films for food preservation

## Abstract

Biodegradable starch-based films often suffer from insufficient mechanical strength, which limits their practical applications. To enhance film performance, this study optimized the preparation of composite thermoplastic starch (CTPS) films composed of corn starch, sorbitol, whey protein isolate (WPI), and gallic acid (GA). The optimized formulation—0.074 g/mL corn starch, 47.5% sorbitol, 5.6% WPI, and 2.0 mg/mL GA—yielded films with a tensile strength of 3.11 ± 0.31 MPa and an elongation at break of 43.35 ± 0.69%, achieving a desirable balance between flexibility and strength. Mechanistic investigations using in situ Fourier-transform infrared spectroscopy (FTIR), low-field nuclear magnetic resonance (LF-NMR), confocal laser scanning microscopy (CLSM), and molecular docking revealed a ternary interaction system among starch, WPI, and GA. These components primarily interacted through hydrogen bonding and van der Waals forces. Such non-covalent interactions enhanced the short-range molecular ordering of the starch matrix, stabilized the secondary structure of WPI, and facilitated water redistribution during film formation. The resulting interaction network among starch, proteins, and polyphenols significantly improved the mechanical properties and antioxidant capacity of the CTPS films.

## 1. Introduction

As the predominant material in food packaging, plastics pose considerable environmental challenges owing to their persistent degradation. The extensive use of plastic packaging has led to notable ecological consequences [1], thereby imposing significant environmental costs. In response to this issue, considerable efforts have been devoted to the design of biodegradable and bio-based materials as eco-friendly alternatives to conventional plastics [2]. Particular interest has been directed toward natural polymers, including polysaccharide-based materials (e.g., starch [3], cellulose [4], chitosan [5]) and protein-based systems (e.g., collagen [6], gelatin [7], soy protein isolate [8]) which are increasingly being studied for sustainable packaging applications [9].

Although starch represents a promising sustainable alternative to plastics due to its renewability, biodegradability, and adjustable functional properties, the extensive hydrogen bonding between hydroxyl groups in native starch induces premature decomposition before melting [10,11]. This inherent characteristic results in inadequate processability and limited mechanical strength in pure starch films [12,13,14], thereby constraining their use in edible or active packaging applications.

To address these drawbacks, a common strategy involves the incorporation of polyhydroxy compounds (e.g., sorbitol) as plasticizers, which render starch thermoplastic and thus processable into thermoplastic starch (TPS) [15,16]. Sorbitol improves the processability of thermoplastic starch (TPS) films through interactions between its hydroxyl groups and those of starch. This disrupts intermolecular hydrogen bonding, increases chain mobility, and suppresses retrogradation, thereby enhancing film flexibility, stability, and overall processability [17].

Thermoplastic starch (TPS) films can be functionalized by incorporating active compounds that interact with the food or its surrounding atmosphere. This capability allows them to extend shelf-life and enhance safety, thereby surpassing the limitations of conventional passive packaging [18]. Whey protein isolate (WPI), which consists of macromolecules including α-lactalbumin, β-lactoglobulin, bovine serum albumin, and immunoglobulins [19], possesses outstanding film-forming capacity, elasticity, and lipid barrier properties [20]. These characteristics contribute to improved mechanical integrity in starch-based films. Gallic acid (GA), a natural polyphenol, exhibits strong antioxidant activity along with other bioactivities such as antimicrobial and anti-inflammatory effects. Notably, proteins can covalently bind with polyphenols like GA [21], a process that induces structural modifications in the protein and consequently enhances its functional and bioactive properties, including antioxidant activity [3,22,23,24].

While the introduction of diverse functional components markedly enhances the processing, mechanical, and functional properties of starch-based films, existing studies have largely concentrated on single-component interactions with starch. A comprehensive understanding of the synergistic or competitive effects in multi-component composite systems remains elusive. These mechanisms, governing the macroscopic film performance at the molecular level, are pivotal for driving their practical implementation. Beyond enhancing the processability and mechanical performance of composite biodegradable films [25,26,27], it is crucial to develop a fundamental understanding of the molecular-level interactions among constituents such as starch, proteins, and polyphenols, which govern the overall film properties. However, while studies have extensively examined binary systems involving polyphenols or proteins with starch [28,29], the ternary system comprising all three components has received limited attention. This study utilized corn starch, sorbitol, WPI, and GA as principal materials. Following single-factor experiments, a Box–Behnken design was employed to optimize these four factors through response surface methodology, thereby establishing the optimal preparation parameters. The molecular interactions within the TPS/WPI/GA films were investigated using an integrated approach combining spectroscopic, microscopic, and computational analyses. By elucidating the ternary interactions among starch, whey protein isolate, and gallic acid, this work provides theoretical foundations for the rational design of high-performance biodegradable edible films.

## 2. Materials and Methods

### 2.1. Materials

The following materials were used as received: corn starch (food grade, containing approximately 87% dry solids and 11% moisture; Yantai Shuangta Food Co., Ltd. (Yantai, Shandong Province, China)); sorbitol (98%), absolute ethanol (95%), ABTS (2,2′–azino-bis (3-ethylbenzothiazoline- 6-sulfonic acid) diammonium salt, 98%), DPPH (2,2–diphenyl–1-picrylhydrazyl, 96%), whey protein isolate (80%), gallic acid (99%), disodium hydrogen phosphate (98%), sodium dihydrogen phosphate (98%), rhodamine B (≥99%), and FITC (fluorescein isothiocyanate, ≥95%) from Shanghai Macklin Biochemical Technology Co., Ltd. (Shanghai, China); and potassium bromide from Tianjin Komiou Chemical Reagent Co., Ltd. (Tianjin, China). All reagents were of analytical or spectroscopic grade.

### 2.2. Instruments

The experimental setup utilized the following equipment: BG12C ultrasonic generator (Guangzhou Bangjie Electronic Products Co., Ltd., Guangzhou, China); TA-XT plus texture analyzer (Stable Micro Systems Ltd., Godalming, UK); SPX-250-II constant climate chamber (Shanghai Xinmiao Medical Device Manufacturing Co., Ltd., Shanghai, China); ReactIR 15 in situ FTIR spectrometer (Mettler-Toledo International Inc. Columbus, OH, USA); IIVTMR20-101 low-field NMR analyzer (Suzhou Niumag Analytical Instrument Co., Ltd., Suzhou, China); UV7600 dual-beam UV-Vis spectrophotometer (Shanghai Xipu Instrument Co., Ltd., Shanghai, China); and FV3000 confocal laser scanning microscope (Olympus Corporation, Tokyo, Japan).

### 2.3. Preparation of Thermoplastic Starch Films

The preparation of thermoplastic starch films involved the following procedure: Corn starch (0.023–0.092 g), sorbitol (25–45% *w*/*w*, dry starch basis), and whey protein isolate (2–10% *w*/*w*, dry starch basis) were dissolved in 130 mL deionized water. After 5 min of ultrasonication for homogenization, the suspension was heated in a water bath with mechanical stirring. The temperature was raised from 40 °C to 95 °C at a heating rate of 10 °C per 10 min, followed by isothermal stirring at 95 °C for 25 min, with continuous stirring at 300 rpm throughout both the heating and isothermal stages. Subsequently, gallic acid (0.5–2.5 mg/mL) was incorporated and the mixture was stirred at 95 °C for 10 min at 400 rpm. Upon reaction completion, the mixture was ultrasonically treated for 5 min and cast into square Petri dishes (10 cm × 10 cm). The cast samples were dried in an environmental chamber at 35 °C and 55% relative humidity for 12 h, followed by 10 min of cooling at room temperature prior to demolding [30,31,32,33,34]. Following demolding, selected films were immediately trimmed for tensile strength and elongation at break testing, while the remainder were sealed in bags and stored under the same controlled conditions.

To ensure accuracy across all analyses, film thickness was precisely controlled by gravimetrically casting a defined mass (30.0 ± 0.50 g) of the fully reacted composite thermoplastic starch solution into each Petri dish. This approach minimized thickness variations, which was critical for obtaining reproducible mechanical and barrier properties.

### 2.4. Effects of Corn Starch, Sorbitol, Whey Protein, and Gallic Acid on the Properties of Composite Thermoplastic Starch Films

Using the procedure outlined in Section 2.3, film formulations were prepared by individually varying four key parameters: starch concentration (0.023, 0.046, 0.069, 0.085, 0.092 g/mL), sorbitol level (25%, 30%, 35%, 40%, 45% *w*/*w*, dry starch basis), WPI content (2%, 4%, 6%, 8%, 10% *w*/*w*, dry starch basis), and the concentration of gallic acid (0.5, 1.0, 1.5, 2.0, and 2.5 mg/mL). The tensile strength (TS) and elongation at break (E) of the resulting thermoplastic starch composite films were subsequently evaluated for each formulation.

### 2.5. Optimization of Preparation Conditions for Composite Thermoplastic Starch Films

The formulation of composite thermoplastic starch (CTPS) films was optimized using a Box–Behnken experimental design (BBD) with four independent factors at three levels each, as summarized in Table 1. The design, implemented in Design—Expert 13, defined the following independent variables: starch concentration (A), sorbitol content (B), WPI content (C), and gallic acid concentration (D). The corresponding response variables comprised tensile strength (TS), elongation at break (E). The experimental design consisted of 29 runs incorporating five center points for assessing experimental error and ensuring model repeatability. To mitigate potential systematic errors, the run sequence was fully randomized. The model’s goodness-of-fit was assessed through the coefficient of determination (R^2^), adjusted R^2^, predicted R^2^, and analysis of variance (ANOVA), along with a lack-of-fit test. A non-significant lack-of-fit result (*p* > 0.05) confirmed the model’s adequate predictive capability. Triplicate measurements were performed for each experimental condition, with averaged values employed in subsequent analyses to guarantee data reliability.

### 2.6. Determination of Tensile Strength and Elongation at Break

In accordance with the ASTM D882-12 guideline [35], tensile tests were performed on the starch film strips using a universal testing machine to determine their tensile strength and elongation at break.

For tensile strength measurement, five points on each TPS film were randomly selected, and thickness was measured using a digital micrometer (accuracy: 0.001 mm; Dongguan Sanliang Precision Measuring Instrument Co., Ltd., Dongguan, China). The average value was recorded as d. Film samples were cut into rectangular strips (5 cm × 1 cm) and tested using a texture analyzer. The test conditions were pre-test speed 1 mm/s, test speed 2 mm/s, and post-test speed 10 mm/s. The texture analyzer probe was A/TG. The average value was calculated, and tensile strength (TS) was determined using Equation (1):

**TS = F/(d × W)** (1)

where TS is the tensile strength (MPa), F is the maximum force at break (N), d is the film thickness (mm), and W is the film width (mm).

For elongation at break, five film strips (5 cm × 1 cm) were tested using a TA-XT plus texture analyzer (Stable Micro Systems Ltd., Godalming, UK) with the same speed parameters. The texture analyzer probe was A/TG. The elongation at break (E%) was calculated using Equation (2):

**E (%) = [(L − L_0_)/L_0_] × 100** (2)

where E is the elongation at break (%), L is the final length of the film (mm), and L0 is the initial length of the film (mm).

### 2.7. In Situ Fourier Transform Infrared (FTIR) Spectroscopy

FTIR analysis was performed using a ReactIR 15 (Mettler-Toledo International Inc. Columbus, OH, USA) in situ spectrometer fitted with a DiComp diamond probe and a 9.5 mm × 1.5 m silver halide (AgX) fiber optic interface (Instrument S/N: 21540; Probe S/N: 21497). The probe was positioned 0.5 cm below the liquid surface and maintained under the following acquisition parameters: spectral range 3000–650 cm^−1^, 16 scans at 4 cm^−1^ resolution, with a 30 s collection time. Prior to detection, background spectra were acquired in both air and solvent with corresponding vapor phase corrections. Spectral collection during the heating process was initiated only after the probe achieved thermal equilibrium with the sample. Before data acquisition, background spectra were recorded for both air and solvent, followed by gas-phase correction. During the heating process, spectral collection was performed only after thermal equilibrium between the probe and the sample had been reached to ensure measurement accuracy.

### 2.8. Confocal Laser Scanning Microscopy (CLSM)

Imaging was carried out on an Olympus FV3000 laser scanning confocal microscope (Olympus Corporation, Tokyo, Japan) equipped with a 40× dry objective (numerical aperture 0.95). The pinhole diameter was uniformly set to 213 μm across all channels to improve signal intensity and image brightness while maintaining effective exclusion of out-of-focus light. Image acquisition was restricted to the XY plane without z-stack scanning. Consistent instrumental conditions (laser power, detector gain, and offset) were applied to all samples to guarantee result comparability.

For staining, fluorescein isothiocyanate (FITC) was dissolved in ultrapure water at 1 mg/mL, and rhodamine B was prepared in ultrapure water at 1 mg/mL. A mixture of 1 mL film-forming solution was supplemented with 100 μL FITC and 60 μL rhodamine B, vortexed thoroughly, and used to stain starch and protein, respectively. Measurement conditions: starch—excitation wavelength 559 nm, emission wavelength 570–620 nm; protein—excitation wavelength 494 nm, emission wavelength 500–540 nm. Images were acquired in XY scanning mode with a resolution of 1024 × 1024 pixels, corresponding to a field of view of 318.2 × 318.2 μm (0.311 μm/pixel). The scanning speed was 2.0 μs/pixel. Representative images were selected from three independent experiments.

Imaging technique consideration: It should be noted that the addition of gallic acid (GA) is expected to alter the system pH, which may decrease the staining efficacy of FITC for starch. Therefore, for samples containing GA where confocal fluorescence signal was insufficient, transmission microscopy was utilized as an alternative method to obtain clear morphological images. All other samples were analyzed using the confocal microscopy parameters specified above.

### 2.9. Low-Field Nuclear Magnetic Resonance (LF-NMR) Analysis

The fully reacted composite thermoplastic starch solution was cast into Petri dishes with gravimetrically controlled mass, as detailed in Section 2.4, followed by drying in a constant temperature and humidity incubator at 35 °C and 55% relative humidity. Throughout the drying process, transverse relaxation time (T_2_) measurements were collected at 1 h intervals using a low-field NMR analyzer (IIVTMR20-101), with inter-sample mass differences not exceeding 2 mg. CPMG acquisition parameters were: P1 (90° pulse width) = 3.2 μs, P2 (180° pulse width) = 6.0 μs, SW (sampling frequency) = 333.33 kHz, TW (repetition delay) = 8500 ms, NECH (number of echoes) = 18,000, TD (number of sampling points) = 1,750,038, TE (echo time) = 0.35 ms, and NS (number of slices) = 32. All measurements were performed at room temperature (25 ± 2 °C).

### 2.10. Molecular Docking Simulation

Molecular docking simulations were conducted using AutoDockTools 1.5.6 equipped with the AutoDock 4.2 engine, along with Discovery Studio 2019 Client. The three-dimensional structure of corn starch was modeled using maltotriose (CID: 192826) as a representative oligosaccharide. Gallic acid (CID: 370) and β-lactoglobulin (PDB: 1BEB) structures were retrieved from the PubChem and RCSB PDB databases, respectively. The selection of β-lactoglobulin, which comprises 45–60% of whey protein, served as a representative model for WPI in this study. Prior to molecular docking, the ligand structures (maltotriose and gallic acid) underwent energy minimization employing the CHARMm force field within Discovery Studio 2019 Client. The docking simulation was centered on β-lactoglobulin as the target protein. A spherical search region of 9 Å diameter was defined in Discovery Studio around the predicted binding site. Automatically positioned by the software according to potential active sites, this sphere encompassed the most energetically favorable ligand-binding cavity, thereby implementing a directed docking strategy. The molecular docking simulations employed the default LibDock algorithm within Discovery Studio 2019, utilizing a rigid receptor protocol where the protein structure remained fixed while all rotatable bonds in the ligands were permitted to rotate. All search parameters— including the number of conformations to generate, population parameters, and scoring functions—retained their default software-defined values. For each ligand, ten docking conformations were generated and subsequently ranked by their LibDock scores. The conformation exhibiting the lowest interaction energy was identified as the most favorable binding mode. Finally, the protein-ligand interactions were visualized and analyzed through both 2D and 3D representations available in Discovery Studio.

### 2.11. Statistical

Statistical analyses were performed using SPSS 26.0 (IBM Corp., Armonk, NY, USA). One-way analysis of variance (ANOVA) was conducted, and post hoc comparisons were performed using Duncan’s and least significant difference (LSD) tests at a 95% confidence level (*p* < 0.05). Homogeneity of variance was verified using Levene’s test. Graphical illustrations and correlation analyses were carried out using Origin 2021 (OriginLab Corp., Northampton, MA, USA), and data processing was performed with Microsoft Excel 2016. All experiments were conducted in triplicate (*n* = 3), and results are expressed as mean ± standard deviation (SD). It is acknowledged that while *n* = 3 is a common practice, this sample size may impose constraints on the extrapolation of the findings, a point further considered in the Discussion.

## 3. Results and Discussion

### 3.1. Effect of Starch Content on the Mechanical Properties of Composite Thermoplastic Starch Films

**Figure 1:**
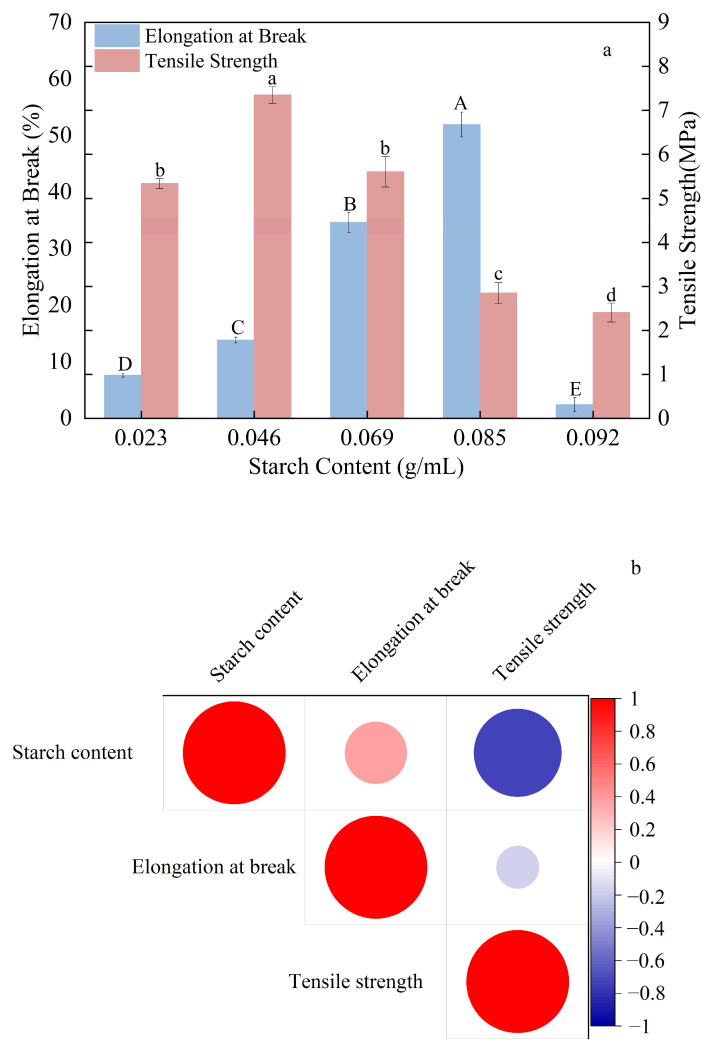
Influence of starch content on the mechanical performance of composite thermoplastic starch (CTPS) films and associated correlation analyses. (**a**) Effects of starch content on both elongation at break and tensile strength; (**b**) Correlations between starch content and respective mechanical properties. (Notes: Different lowercase letters indicate statistically significant differences in tensile strength, while different uppercase letters indicate statistically significant differences in elongation at break (*p* < 0.05)).

Following 12 h drying under controlled conditions (35 °C, 55% RH), the composite thermoplastic starch (CTPS) films were demolded, sectioned according to the specifications in Section 2.6, and subsequently subjected to mechanical property characterization. Figure 1a illustrates that increasing the corn starch concentration from 0.023 g/mL to 0.092 g/mL resulted in an initial increase followed by a decrease in both the elongation at break and tensile strength of the composite thermoplastic starch films. The maximum elongation at break (51.94 ± 2.19%) was achieved at 0.085 g/mL starch, whereas the highest tensile strength (7.34 ± 0.19 MPa) was observed at 0.046 g/mL. During drying at starch concentrations of 0.023–0.069 g/mL, water evaporation promoted a gelatinized starch network, while the higher starch content facilitated closer chain packing, together resulting in a more continuous and compact film structure [36]. As further illustrated in the correlation heatmap (Figure 1b), starch concentration shows a weak positive correlation with elongation at break but a significant negative correlation with tensile strength. This implies that although moderate starch content improves film flexibility [37], excessively high concentrations could lead to reduced structural integrity and strength.

When the starch concentration exceeded 0.046 g/mL, the tensile strength of the composite thermoplastic starch (TPS) film exhibited a declining trend. This reduction may be attributed to the substantial increase in the viscosity of the gelatinized mixture at higher starch concentrations. Under such conditions, a portion of the starch granules may remain incompletely gelatinized because they become encapsulated within the highly viscous matrix, leading to a disruption in the structural continuity of the film. Furthermore, the relative decrease in the water content of the starch film could reduce its plasticization effect, thereby diminishing the flexibility and toughness of the composite film [38].

At very low starch levels (<0.023 g/mL), the film-forming solution exhibited excessive fluidity, making it difficult to cast, and the resulting films were overly thin and fragile, hindering demolding. Conversely, at excessively high concentrations (>0.092 g/mL), the solution became too viscous and lost fluidity, leading to uneven casting and non-uniform film thickness, which complicated property measurements.

Taking these factors into account, a starch addition of 0.069 g/mL was selected for subsequent experiments, as it provided a balanced compromise between mechanical strength, film continuity, and processability.

### 3.2. Effect of Sorbitol on the Formation Mechanism and Mechanical Behavior of Composite Thermoplastic Starch Films

**Figure 2:**
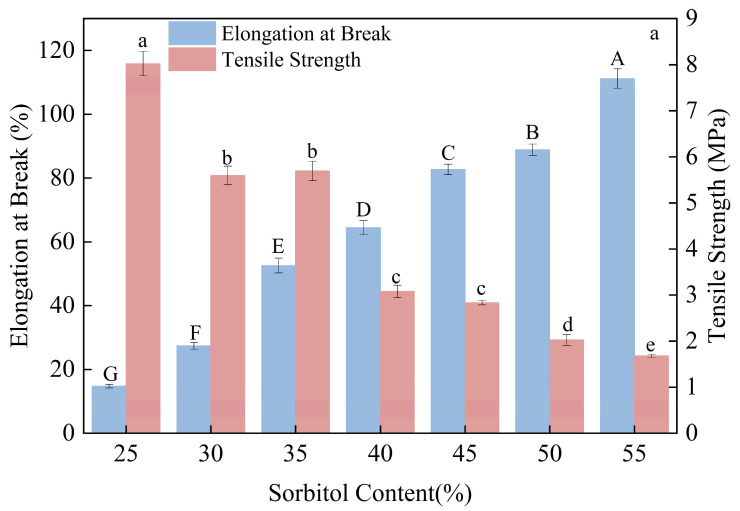

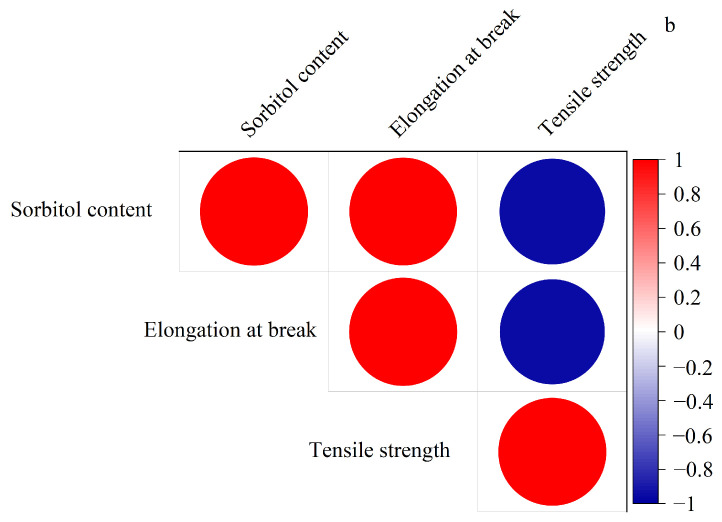
Effects of Different Sorbitol Contents on the Mechanical Properties of Composite Thermoplastic Starch Films Their Correlation Analysis. (**a**), impact of sorbitol content on the elongation at break and the tensile strength of CTPS; (**b**), the correlation analysis of sorbitol content and the elongation at break/the tensile strength of CTPS. (Notes: Different lowercase letters indicate statistically significant differences in tensile strength, while different uppercase letters indicate statistically significant differences in elongation at break (*p* < 0.05)).

Figure 2a illustrates that increasing sorbitol content led to a progressive decline in tensile strength but conversely enhanced elongation at break. These opposing trends were further confirmed in the correlation heatmap (Figure 2b), which revealed a significant negative correlation with tensile strength and a positive correlation with elongation at break.

As a high-molecular-weight plasticizer, sorbitol increases the number of hydroxyl groups per unit volume as its concentration rises, thereby enhancing its capacity to bind water molecules. During the gelatinization process, sorbitol molecules penetrate between starch chains and form hydrogen bonds with hydroxyl groups on the starch backbone. This interaction reduces the availability of hydroxyl groups for inter-chain hydrogen bonding, thereby weakening the binding force between starch molecules and increasing the mobility of macromolecular chains [19]. Consequently, the free volume within the film matrix expands, enhancing molecular slippage and conferring greater elasticity and extensibility to the system, which leads to higher elongation at break [30,36,37].

Considering the overall performance of the films, a sorbitol content of 45% was selected for subsequent experiments.

### 3.3. Effect of Whey Protein Isolate Content on the Mechanical Properties of Composite Thermoplastic Starch Films

**Figure 3:**
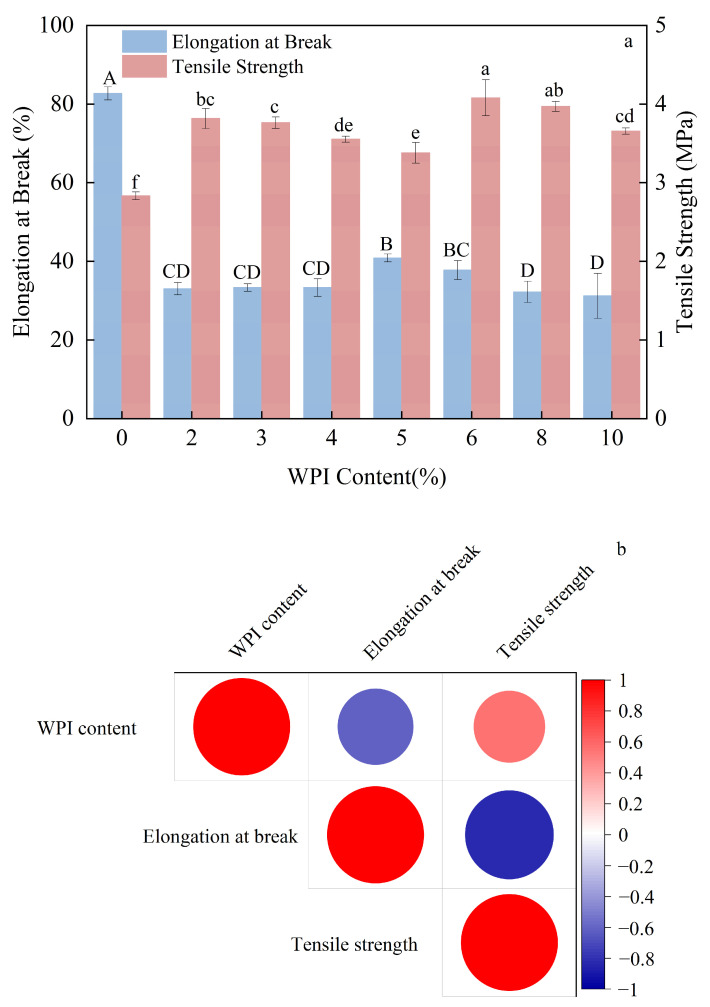
Effects of Different Whey Protein Isolate Contents on the Mechanical Properties of Composite Thermoplastic Starch Films Their Correlation Analysis. (**a**), impact of whey protein content on the elongation at break and the tensile strength of CTPS; (**b**), the correlation analysis of whey protein content and the elongation at break/the tensile strength of CTPS. (Notes: Different lowercase letters indicate statistically significant differences in tensile strength, while different uppercase letters indicate statistically significant differences in elongation at break (*p* < 0.05)).

As shown in Figure 3, the addition of WPI significantly reduced elongation at break while markedly enhancing tensile strength. With increasing WPI content, elongation at break initially increased and then declined, whereas tensile strength exhibited the opposite trend—first decreasing and subsequently rising.

The observed enhancement is likely due to the macromolecular composition of WPI—containing α-lactalbumin, β-lactoglobulin, bovine serum albumin, and immunoglobulins [39]—which enables covalent binding with starch through the Maillard reaction. This reaction between free amino groups in protein side chains and carbonyl groups at starch reducing ends [40] promotes the formation of a denser homogeneous phase [39], thereby improving the film’s tensile strength. Alternatively, the improvement in tensile strength may be attributed to non-covalent interactions between WPI and corn starch, which enhanced the structural ordering of starch and thereby contributed to the mechanical reinforcement of the composite films [41,42]. At moderate WPI concentrations (2–5%), the number of potential binding sites between starch and WPI decreases, and excess WPI may cause phase heterogeneity, leading to a reduction in tensile strength. However, at higher WPI contents (6–10%), cross-linking between protein molecules may occur, further reinforcing the film structure and resulting in an increase in tensile strength.

### 3.4. Effect of Gallic Acid Content on the Mechanical Properties of Composite Thermoplastic Starch Films

**Figure 4:**
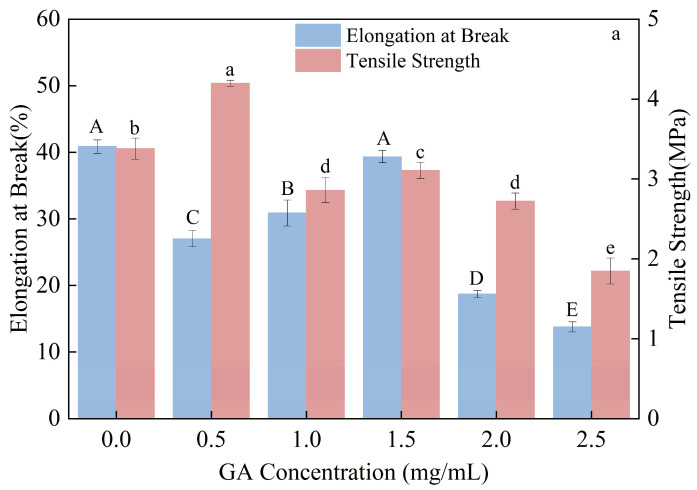

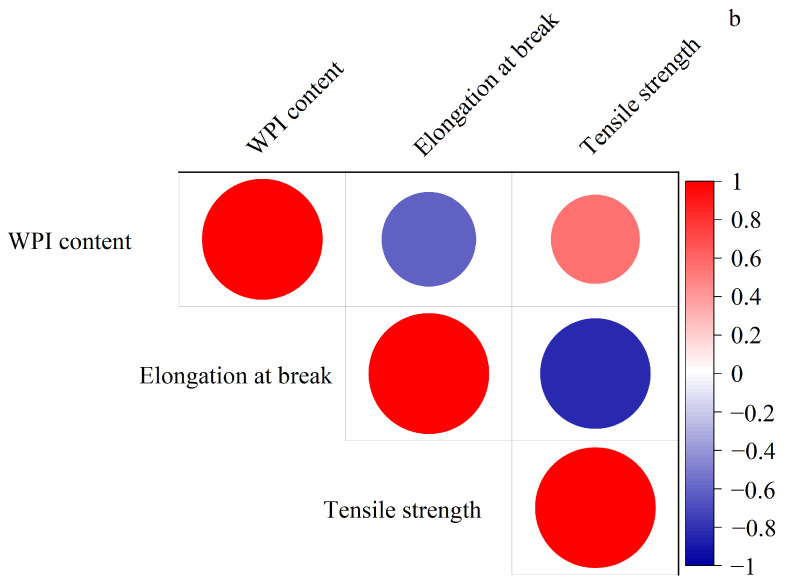
Effects of Different Gallic Acid Contents on the Mechanical Properties of Composite Thermoplastic Starch Films and Their Correlation Analysis. (**a**), impact of gallic acid content on the elongation at break and the tensile strength of CTPS; (**b**), the correlation analysis of gallic acid content and the elongation at break/the tensile strength of CTPS. (Notes: Different lowercase letters indicate statistically significant differences in tensile strength, while different uppercase letters indicate statistically significant differences in elongation at break (*p* < 0.05)).

As shown in Figure 4, the addition of GA caused the elongation at break to follow a fluctuating trend: initially decreasing, then increasing, and finally decreasing again. This behavior may be attributed to the weakening of intra-chain cross-linking and aggregation of starch molecules upon the incorporation of polyphenols, which reduced elongation at break. As GA content increased, polyphenols were able to form hydrogen bonds with water molecules, thereby promoting additional intra-chain cross-linking and aggregation within the starch matrix [42], which enhanced the elongation at break [43,44].

By contrast, tensile strength generally decreased with increasing GA concentration, and correlation analysis revealed a negative relationship between tensile strength and GA content.

### 3.5. Optimization and Analysis of Preparation Conditions for Composite Thermoplastic Starch Films

To further optimize the optimal formulation of thermoplastic starch (TPS) films incorporating both whey protein isolate (WPI) and gallic acid (GA), the amounts of individual components in the composite TPS films were systematically varied based on previous experiments. The experimental design and results are summarized in Table 2. The resistance to fracture under tensile stress is a key mechanical parameter reflecting both the structural stability of the material and its potential for practical applications [45].

Using Design-Expert 13 software, response surface methodology (RSM) was applied to analyze the experimental data. With tensile strength (TS) as the response variable, a quadratic polynomial regression model was obtained as follows:

Y = 3.83 + 0.1942A − 0.2992B + 0.01C − 0.025D + 0.19AB + 0.015AC − 0.2225AD − 0.075BC + 0.0525BD + 0.175CD − 0.4229A^2^ − 0.5104B^2^ − 0.1092C^2^ − 0.3892D^2^ (3)

The analysis of variance (ANOVA) for this model is shown in Table 3.

The model exhibited an F-value of 11.31 with a *p* < 0.05, indicating that the model terms had significant effects on the response variable. The non-significant result of the lack-of-fit test (*p* = 0.7499 > 0.05) demonstrates that the model adequately fits the experimental data. Factors C, D, AC, BC, BD, CD, and C^2^ had *p* > 0.05, indicating that their effects were not statistically significant. In contrast, the interaction terms AB and AD had *p* < 0.05, suggesting significant interactions between starch content and sorbitol, and between starch content and GA. Moreover, the quadratic terms A^2^, B^2^, and D^2^ were highly significant (*p* < 0.05). The relative importance of the factors, based on *p*-values, followed the order: B > A > D > C.

When elongation at break (E) was considered as the response variable, the following quadratic regression model was obtained:

Y = 44.12 − 1.62A + 2.99B − 0.4842C + 1.54D + 0.005AB + 4.12AC + 5.30AD + 2.35BC + 5.25BD + 1.09CD − 4.70A^2^ − 4.25B^2^ − 4.93C^2^ − 4.27D^2^ (4)

The corresponding ANOVA results are presented in Table 4.

The model showed an F-value of 8.38 with a *p* < 0.05, confirming that the model terms significantly influenced the response. The lack-of-fit test yielded a *p* = 0.9177 (>0.05), again suggesting a good model fit. Factors C, AB, BC, BD, and CD were not significant (*p* > 0.05), while AC and AD exhibited significant interaction effects (*p* < 0.05), indicating strong interactions between starch content and WPI, as well as starch content and GA. Furthermore, the quadratic terms A^2^, B^2^, C^2^, and D^2^ were all significant (*p* < 0.05). Based on the significance levels, the influence of the factors on elongation at break was ranked as: B > A > D > C.

The linear term for whey protein isolate (WPI) content (Factor C) was not statistically significant for either tensile strength (*p* = 0.8464; Table 3) or elongation at break (*p* = 0.5097; Table 4). This finding, however, does not imply that WPI lacks influence on the mechanical properties of the composite thermoplastic starch (CTPS) films. Rather, it indicates that the effect of WPI is fundamentally non-linear and contingent upon its interactions with other components in the system. Notably, the highly significant quadratic term for WPI (C^2^) suggests the presence of an optimal concentration range, opposing a simple linear dose–response relationship. This aligns with the trends observed in the single-factor experiments (Section 3.3), where moderate WPI addition enhanced film flexibility, while excessive amounts led to protein aggregation and compromised extensibility. Furthermore, the significance of interaction terms involving WPI, most notably the AC term (starch-WPI interaction), underscores that WPI’s impact on film performance is intrinsically linked to the starch matrix. WPI is proposed to engage in hydrogen bonding and physical entanglement with gelatinized starch chains [41,42]. Consequently, its contribution to the mechanical network becomes pronounced only when evaluated in combination with starch content, not in isolation. Therefore, the lack of significance for the linear term C reflects the complex, non-linear, and interaction-dependent role of WPI within the starch-protein-polyphenol ternary system, not an absence of function.

For tensile strength (TS, Table 3), the significance of the AB (starch-sorbitol) and AD (starch-GA) interactions reveals a synergistic reinforcement mechanism. The AB interaction highlights sorbitol’s dual role: at optimal ratios with starch, it plasticizes effectively to facilitate molecular rearrangement and stress dissipation, whereas imbalance can lead to over-plasticization and network weakening. Concurrently, the AD interaction demonstrates that gallic acid (GA) acts as a secondary crosslinker via hydrogen bonding with the starch matrix, enhancing load-bearing capacity only when integrated with a sufficient starch network. Thus, TS is optimized not by maximizing individual constituents, but by precisely balancing these starch-centric interactions to construct a coherent, reinforced network.

Regarding elongation at break (E, Table 4), the dominant AC (starch-WPI) and AD (starch-GA) interactions unveil a complementary mechanism regulating flexibility. The AC interaction indicates that WPI enhances extensibility through physical entanglement and hydrogen bonding with starch chains, a contribution that is strictly dependent on starch content to avoid protein aggregation. The AD interaction, again pivotal, shows GA modulates flexibility through a balance: moderate levels impart plasticity, while higher levels introduce crosslinking-induced stiffness, a trend consistent with the single-factor experiments on GA content (Section 3.4). Therefore, the ultimate extensibility is a non-linear product of these cooperative interactions, where starch serves as the essential scaffold for both protein and polyphenol to express their functional roles.

In conclusion, we explicitly emphasize that the optimization of the CTPS films’ mechanical properties is predominantly orchestrated through these critical binary interactions involving starch. They are the strongest optimization mechanism because they directly control the nano/micro-scale assembly of the ternary components. This interaction-driven paradigm, effectively captured by RSM, explains why the system’s performance cannot be predicted by linear composition effects alone, highlighting the sophistication of the developed composite material.

According to the optimization analysis conducted with Design-Expert 13, the predicted optimal formulation for CTPS films was 0.074 g/mL starch, 47.47%, sorbitol, 5.63% WPI, and 2.0 mg/mL GA. Considering the practical limitations of experimental operations and potential errors, the final optimized formulation was adjusted to 0.074 g/mL starch, 47.5% sorbitol, 5.6% WPI, and 2.0 mg/mL GA. Under these conditions, triplicate experiments were performed, and the average mechanical properties of the films were determined to be TS = 3.11 ± 0.31 MPa and E = 43.35 ± 0.69%. These results confirmed that the regression models provided a reliable fit to the experimental data.

### 3.6. In Situ FTIR Analysis

**Figure 5:**
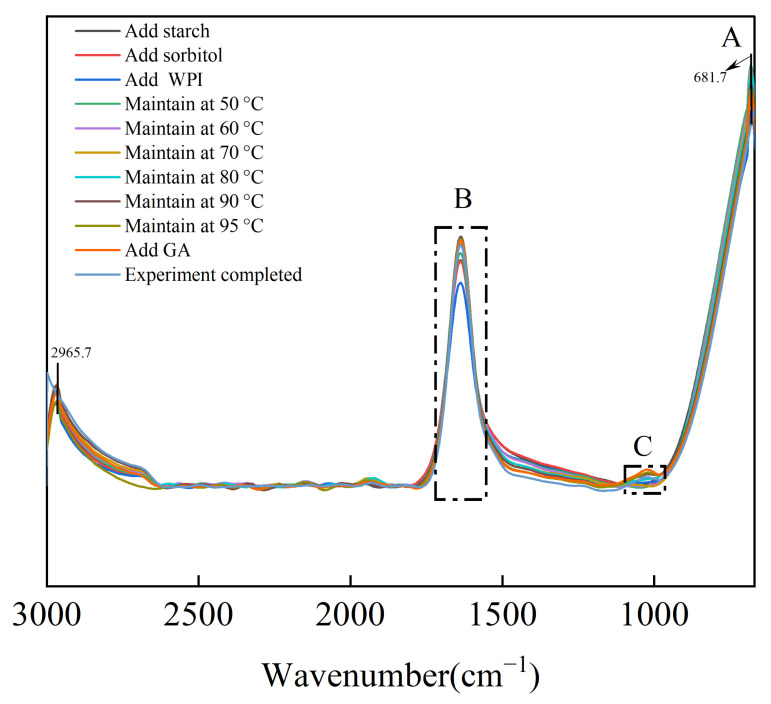

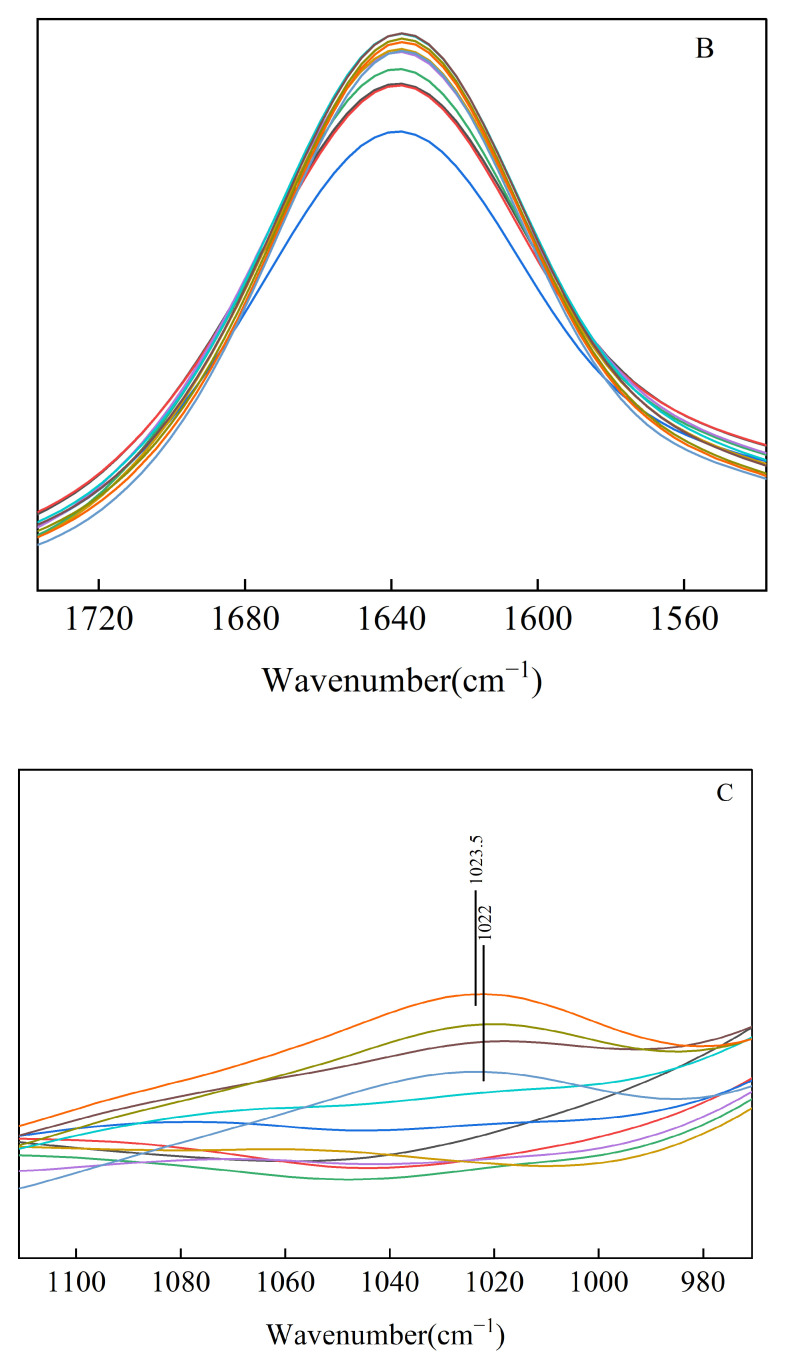
In situ Fourier-transform infrared spectroscopy monitoring during the preparation of composite thermoplastic starch films. (Samples were taken at each of the following stages: after the addition of corn starch, after the addition of sorbitol, after the addition of whey protein isolate, after the addition of gallic acid, at every 10 °C temperature increment, and upon completion of the experiment). (**A**) In situ FTIR spectra recorded during the entire reaction process. (**B**) Enlarged in situ FTIR spectra in the wavenumber range of 1720–1560 cm^−1^ showing the evolution of characteristic absorption bands during the reaction. (**C**) Enlarged in situ FTIR spectra in the wavenumber range of 980–1100 cm^−1^ illustrating the changes in characteristic absorption bands as the reaction proceeds.

The in situ FTIR spectra (Figure 5A) display a peak near 2965 cm^−1^ assigned to –CH_2_ antisymmetric stretching, and a band at 681 cm^−1^ associated with C–C stretching and starch skeletal vibrations. Figure 5B shows a pronounced absorption at 1640 cm^−1^, indicative of O–H bending and water located in amorphous regions of starch [39,46]. In the early heating phase, the intensity of the 1640 cm^−1^ band rose, indicating the formation of additional non-covalent hydrogen bonds involving hydroxyl groups from water or plasticizers and starch. As heating proceeded, the band intensity decreased, which may be ascribed to rearrangement of amorphous regions and loss of water. These spectral changes correspond to dehydration of starch granules, alterations in structural stability, and reconfiguration of plasticizers. This spectral region additionally contains contributions from N–H bending vibrations and coincides with the C=O stretching vibration of the amide I band.

The spectral region shown in Figure 5C (950 cm^−1^–1180 cm^−1^) represents the fingerprint zone characteristic of polysaccharides, which comprises highly coupled vibrations of C–O, C–O–C, and C–C stretching along with C–O–H bending modes [47]. A distinct absorption band near 1020 cm^−1^, assigned to C–O stretching and C–O–H bending, gradually emerged during the reaction and shifted from 1023.5 cm^−1^ to approximately 1022 cm^−1^. This low-wavenumber shift suggests the incorporation of sorbitol, WPI, and GA into the starch matrix and the formation of new hydrogen bonds with functional groups such as O–H, C–O–H, and C–O–C [42,47]. Importantly, no peaks disappeared or newly appeared, supporting that the corn starch–WPI–GA complex was formed via non-covalent interactions rather than covalent bonding.

All DO values were calculated from triplicate measurements and are reported as mean ± standard deviation. Standard deviations were rounded to two decimal places, which resulted in values of 0.00 or 0.01 for some conditions.

In FTIR analysis, the absorption band at approximately 1022 cm^−1^ is characteristic of the amorphous domains, whereas the band at 1047 cm^−1^ represents the crystalline domains, reflecting the structural ordering of starch aggregates [48]. The short-range molecular order (DO) of starch is quantified by the intensity ratio of the peaks at 1047 cm^−1^ and 1022 cm^−1^ (I_1047_/I_1022_). An increase in the DO value indicates enhanced short-range structural ordering in the system [49]. The in situ FTIR spectra (Figure 5A) display a peak near 2965 cm^−1^ assigned to –CH_2_ antisymmetric stretching, and a band at 681 cm^−1^ associated with C–C stretching and starch skeletal vibrations. Figure 5B shows a pronounced absorption at 1640 cm^−1^, indicative of O–H bending and water located in amorphous regions of starch [40,47]. In the early heating phase, the intensity of the 1640 cm^−1^ band rose, indicating the formation of additional non-covalent hydrogen bonds involving hydroxyl groups from water or plasticizers and starch. As heating proceeded, the band intensity decreased, which may be ascribed to rearrangement of amorphous regions and loss of water. These spectral changes correspond to dehydration of starch granules, alterations in structural stability, and reconfiguration of plasticizers. This spectral region additionally contains contributions from N–H bending vibrations and coincides with the C=O stretching vibration of the amide I band.

The spectral region shown in Figure 5C (950 cm^−1^–1180 cm^−1^) represents the fingerprint zone characteristic of polysaccharides, which comprises highly coupled vibrations of C–O, C–O–C, and C–C stretching along with C–O–H bending modes [48]. A distinct absorption band near 1020 cm^−1^, assigned to C–O stretching and C–O–H bending, gradually emerged during the reaction and shifted from 1023.5 cm^−1^ to approximately 1022 cm^−1^. This low-wavenumber shift suggests the incorporation of sorbitol, WPI, and GA into the starch matrix and the formation of new hydrogen bonds with functional groups such as O–H, C–O–H, and C–O–C [43,48]. Importantly, no peaks disappeared or newly appeared, supporting that the corn starch–WPI–GA complex was formed via non-covalent interactions rather than covalent bonding.

As shown in Table 5, the incorporation of WPI significantly enhanced the DO value, which can be attributed to the formation of hydrogen bonds between WPI and starch. This conclusion is further supported by the increase in peak intensity observed in Figure 5A after the addition of WPI. With increasing temperature, the DO value first increased and then decreased, consistent with the trend shown in Figure 5C. With further gelatinization, starch granules absorbed water, swelled, and ruptured, leading to the disruption of double-helical structures and a consequent decrease in short-range order, as reflected by the reduced DO values. Notably, the incorporation of gallic acid (GA) significantly increased the short-range molecular order of the system. The final DO value (0.95 ± 0.01) was significantly higher than that of native starch (0.90 ± 0.00, *p* < 0.05), indicating that GA effectively promoted the reorganization and stabilization of starch short-range ordered structures during film formation. These results suggest that GA facilitates non-covalent interactions with starch hydroxyl groups, thereby enabling the formation of a tighter composite structure with WPI [42]. Specifically, GA can associate with terminal disordered starch chains via phenolic hydroxyl groups, promoting the formation of helical complexes and localized ordered aggregates.

**Figure 6:**
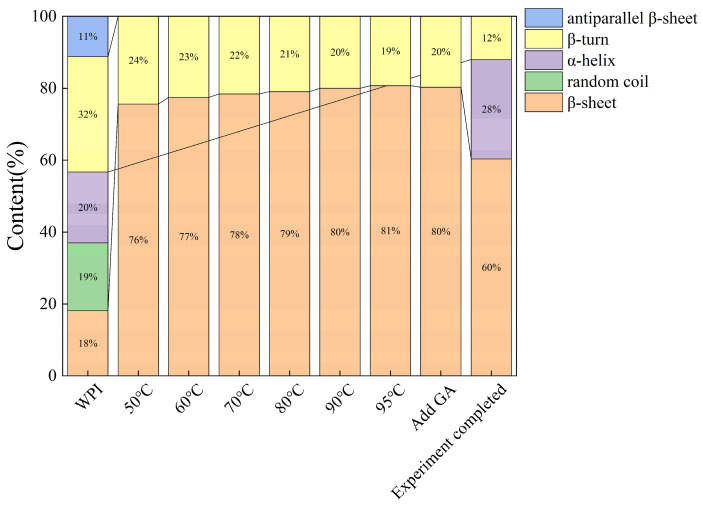
Evolution of protein secondary structure during the reaction process.

Analysis of the secondary structural evolution in Figure 6 reveals that the conformation of native WPI at room temperature is dominated by α-helix, β-turn, and β-sheet. Heating from 50 °C to 95 °C reduced the α-helix content from 20% to 0% and markedly increased the β-sheet content from 18% to 80%, demonstrating heat-induced denaturation and structural reorganization. Under quiescent heating conditions, the unfolding of native α-helices and intramolecular β-sheets into random domains preceded the formation of intermolecular β-sheets, thereby driving aggregation [41]. Upon incorporation of GA, cross-linking via hydrogen bonds and other non-covalent interactions with WPI likely allows GA to associate with structural motifs such as random coils or hydrophobic regions on the protein [42]. This association may confer a shielding effect that mitigates unfolding, favoring the recovery of α-helix content to 28%. Another plausible mechanism is that GA promotes the assembly of interprotein cross-linked complexes, which restricts the linear stacking of β-sheets and consequently reduces their overall abundance [43]. Such structural modulation is also indicative of GA-directed changes in protein secondary structure [50]. The concurrent increase in α-helix and β-sheet content contributed to greater mechanical strength of the films at the expense of ductility and flexibility. This observation aligns with the work of Zhao et al. [51] on the stability of ternary polysaccharide-protein-polyphenol systems. Furthermore, it corroborates the trends identified in our single-factor experiments: a positive correlation between WPI concentration and tensile strength, and, within a defined range, between GA concentration and elongation at break.

### 3.7. Confocal Laser Scanning Microscopy

**Figure 7:**
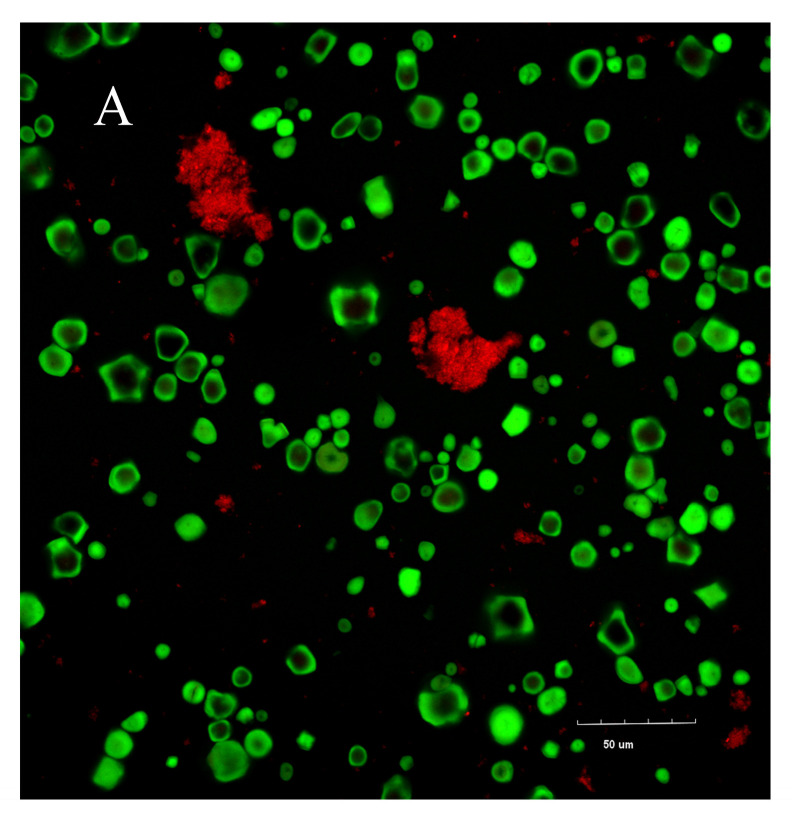

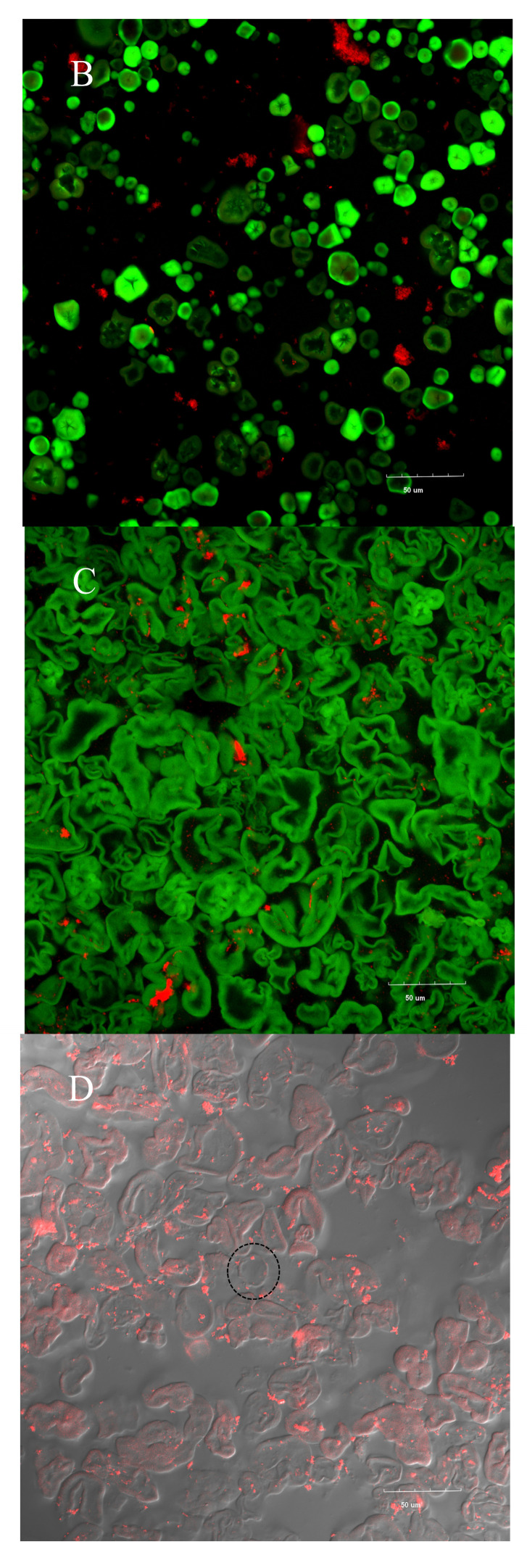

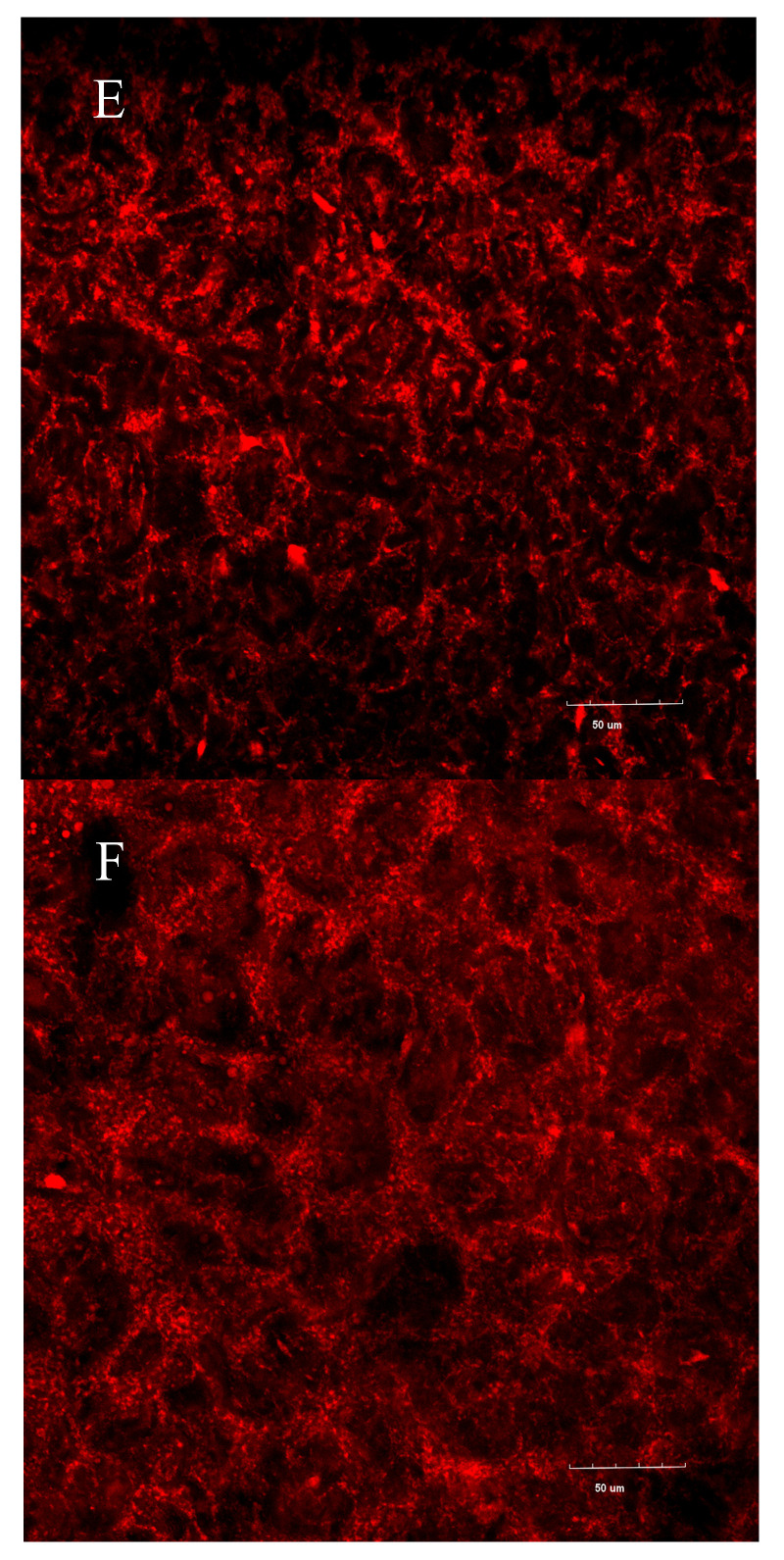
Confocal laser scanning microscopy results: (**A**) complete mixing of the system at 40 °C; (**B**) onset of starch gelatinization at 60 °C; (**C**) complete starch gelatinization at 95 °C; (**D**) addition of gallic acid at 95 °C (starch granules indicated within the circle); (**E**) protein distribution after 5 h of drying; (**F**) protein distribution after 5.5 h of drying.

As shown in Figure 7, prior to the addition of GA, starch granules gradually swelled and ruptured with increasing temperature, allowing WPI to penetrate into the interior of the starch granules. Following GA addition, the altered system pH diminished FITC staining efficiency (as noted in Section 2.8). Accordingly, analysis by transmission microscopy revealed that GA promoted a more refined and uniform dispersion of WPI throughout the matrix (Figure 7D), owing to hydrogen bonding interactions among GA, starch, and WPI. This finding aligns with the enhanced short-range structural ordering reported in Section 3.3 following GA incorporation. Additionally, certain swollen starch granules exhibited a tendency to aggregate in the presence of GA, which may be attributed to cooperative interactions between GA and leached amylose chains [52].

During the drying process (Figure 7E,F), a network-like structure of WPI became evident between 5 h and 5.5 h, with the structure at 5.5 h appearing more regular and complete. This phenomenon may be explained by the reduction in free water content during this period, which promoted intermolecular interactions among WPI molecules, thereby leading to the formation of an ordered network-like distribution.

### 3.8. Low-Field Nuclear Magnetic Resonance Analysis

The transverse relaxation time (T_2_) obtained from low-field nuclear magnetic resonance (LF-NMR) is closely associated with the mobility of hydrogen-bearing fluids. Protons in bound water exhibit restricted mobility and consequently shorter T_2_ relaxation times, whereas those in free water possess greater mobility and higher degrees of freedom, leading to longer T_2_ relaxation times. Therefore, the distribution of relaxation times can be used to distinguish and quantify the fractions of free water, immobilized water, and bound water in a sample and to track their dynamic changes during processing [53].

**Figure 8:**
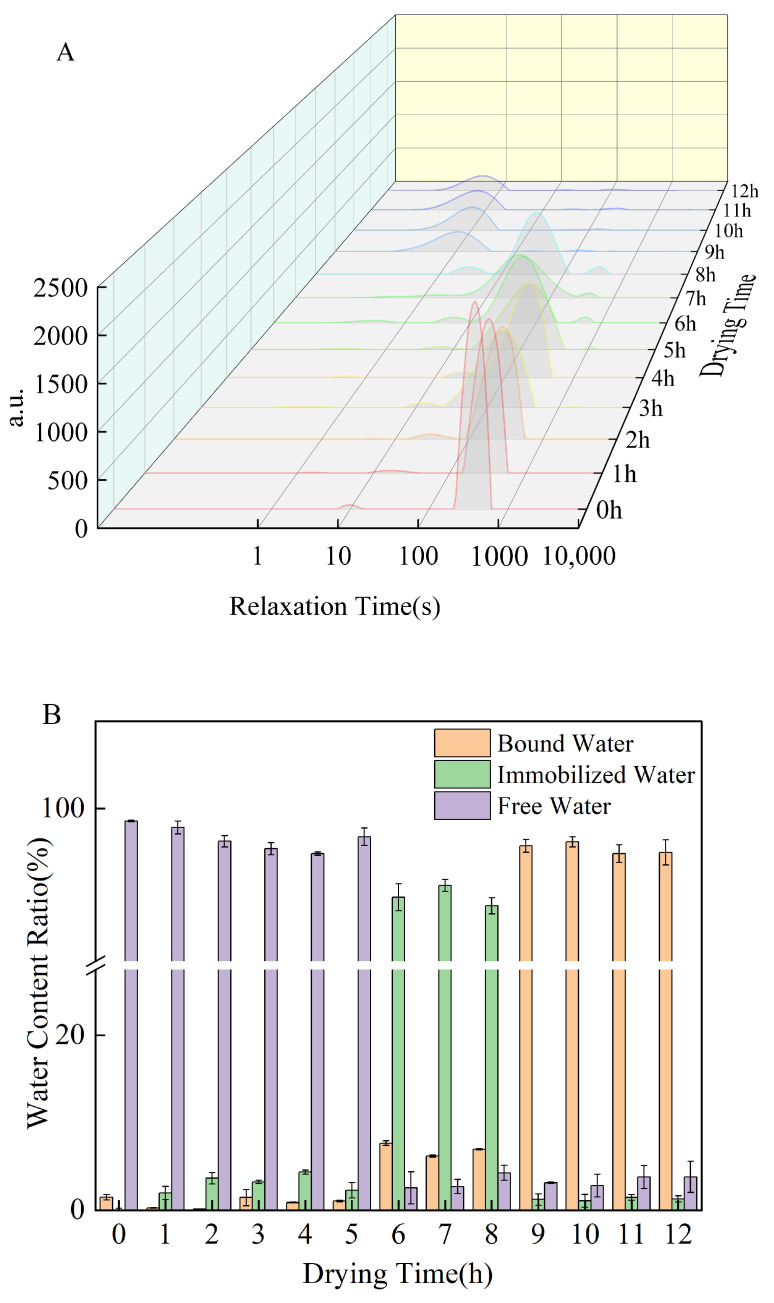
(**A**) T_2_ relaxation time inversion spectra of composite thermoplastic starch films at different drying times; (**B**) Moisture distribution profiles of composite thermoplastic starch films at different drying times.

The T_2_ relaxation times of thermoplastic starch (TPS) films at different drying stages were collected using the Carr–Purcell–Meiboom–Gill (CPMG) sequence. As shown in Figure 8, three distinct peaks were observed during the drying process, corresponding to bound water (T_21_: 0 ms–10 ms), immobilized water (T_22_: 10 ms–100 ms), and free water (T_23_: >100 ms) [15]. The results revealed that water in the TPS system was dominated by free water throughout the drying process, consistently accounting for more than 90% of the total water content.

During drying, the T_2_ relaxation spectra exhibited a progressive shift toward shorter relaxation times, accompanied by a continuous reduction in peak areas, indicating a gradual restriction of water mobility within the starch-based film matrix. Based on the evolution of peak positions and relative peak areas (Table 6), the drying process can be divided into three distinct stages characterized by different dominant water states.

In the early drying stage (0–1 h), free water (A23) was the dominant fraction, accounting for over 97% of the total water at t = 0 h. Due to its weak interactions with the polymer matrix, free water protons were the least constrained and therefore the most readily removed, resulting in a rapid decrease in A23 and a pronounced leftward shift of the corresponding T_2_ peak.

In the intermediate drying stage (2–6 h), the depletion of free water led to a relative increase in the proportion of immobilized and bound water. During this stage, water molecules experienced progressively stronger intermolecular interactions with starch, sorbitol, WPI, and GA through hydrogen bonding and physical confinement, as reflected by shorter relaxation times and the redistribution of peak areas.

In the late drying stage (≥7 h), bound water (A21) became the predominant water fraction. For instance, at t = 10 h, A21 accounted for 96.35% of the total water content, whereas free water was reduced to a negligible level. This transition indicates that the remaining water molecules were strongly associated with the polymer network, consistent with the formation of a dense and stabilized film structure. The relatively minor variation in A23 during the final drying stage suggests that most removable free water had already been eliminated, and the system approached a quasi-equilibrium state dominated by bound water.

Overall, the dramatic transition from free water to bound water during drying provides strong evidence for the progressive establishment of intermolecular interactions and structural consolidation in the composite starch-based films, which is a critical prerequisite for effective film formation [53].

### 3.9. Analysis of Molecular Docking Simulation Results

**Figure 9:**
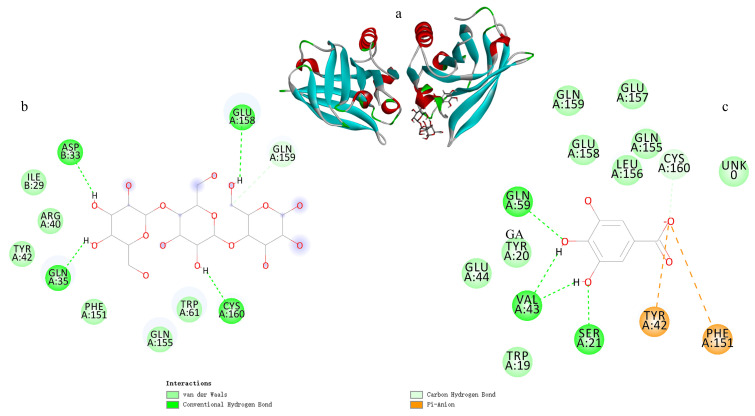
Molecular docking of corn starch and GA with WPI (**a**): overall docking results; (**b**): 2D interactions of starch with WPI; (**c**): 2D interactions of GA with WPI.

As shown in Figure 9b, maize starch interacted with WPI at residues ASP33, GLU158, GLN159, CYS160, and GLN35. Among these, ASP33, GLU158, CYS160, and GLN35 formed hydrogen bonds with hydroxyl groups in the starch structure, while GLN159 interacted through van der Waals forces. These docking results are in good agreement with the in situ infrared spectroscopy findings.

As illustrated in Figure 9c, GA interacted with WPI through residues GLN59, CYS160, PHE151, TYR42, SER21, and VAL43. Specifically, GLN59, SER21, and VAL43 formed hydrogen bonds with GA; CYS160 interacted via van der Waals forces; while PHE151 and TYR42 participated in π-anion interactions with GA. The π-anion interaction, a type of electrostatic interaction, is known to enhance the stability of protein–ligand complexes [54,55].

In summary, the interactions among maize starch, WPI, and GA were primarily governed by a combination of non-covalent forces, including van der Waals interactions, hydrogen bonding, and electrostatic interactions. This conclusion is highly consistent with the in situ infrared spectroscopy analysis, thereby further substantiating the binding mechanism within the thermoplastic starch system composed of maize starch, WPI, and GA.

### 3.10. Study Limitations and Future Perspectives

It is important to consider the limitations of this study, foremost among which is the use of three experimental replicates (*n* = 3). Despite aligning with common methodological standards, this sample size can impact the precision and broader applicability of the results. To address this, subsequent investigations involving larger sample cohorts would be valuable to confirm and extend the present findings.

## 4. Conclusions

In this study, composite thermoplastic starch (CTPS) films were successfully prepared using corn starch, sorbitol, whey protein isolate (WPI), and gallic acid (GA) as raw materials. Through formulation optimization, the CTPS films with the best performance exhibited a tensile strength of 3.11 ± 0.31 MPa and an elongation at break of 43.35 ± 0.69%. The optimal composition was determined as 0.074 g/mL starch, 47.5% sorbitol (relative to starch weight), 5.6% WPI, and 2.0 mg/mL GA. The film-forming mechanism of starch-based packaging materials was attributed to the synergistic interactions among starch, protein, and polyphenols within the ternary system. These interactions were predominantly governed by physical cross-linking mediated by hydrogen bonding rather than covalent chemical reactions. Both whey protein isolate (WPI) and gallic acid (GA) significantly enhanced the short-range molecular order of the starch matrix. Notably, GA incorporation resulted in a statistically significant increase in the degree of order (DO) of starch, with the final DO value (0.95 ± 0.01) being significantly higher than that of native starch (0.90 ± 0.00, *p* < 0.05), while simultaneously contributing to the partial restoration of protein helical structures, thereby improving overall film stability.

During film formation and drying, free water was rapidly depleted, whereas bound water gradually increased and ultimately became the dominant water fraction after drying. This pronounced transition from free water to bound water reflects the progressive strengthening of intermolecular interactions and the consolidation of a dense polymer network, which is crucial for the structural integrity and stability of the resulting films. Meanwhile, WPI progressively assembled into a stable network structure during drying, as confirmed by confocal laser scanning microscopy.

At the molecular level, corn starch and WPI primarily interacted through hydrogen bonding and van der Waals forces, whereas GA–WPI interactions involved multiple noncovalent interactions, including hydrogen bonding, van der Waals forces, and the specific π–anion electrostatic interaction revealed by molecular docking. Overall, this work not only provides mechanistic insights into the film-forming process of starch–protein–polyphenol systems by clearly distinguishing these interaction mechanisms, but also establishes a solid theoretical foundation for the rational design of stable, sustainable, and functional starch-based packaging materials.

## Figures and Tables

**Table 1:** Experimental design of response surface methodology.

Level	Factors			
	Starch Content(A)/ g/mL	Sorbitol Content (*w*/*w*) (B)/ %	Whey Protein Isolate Content (*w*/*w*) (C)/ %	Gallic Acid Content (D)/ mg/mL
−1	0.062	40	4	1.0
0	0.069	45	5	1.5
1	0.077	50	6	2.0

**Table 2:** Response surface design and experimental results.

Run No.	Starch Content (g/mL)	Sorbitol Content (*w*/*w* %)	WPI Content (*w*/*w* %)	GA Concentration (mg/mL)	Tensile Strength (MPa)	Elongation at Break (%)
1	0.069	45	6	1.0	3.23 ± 0.02 ^fghi^	31.14 ± 0.17 ^ijkl^
2	0.077	45	6	1.5	3.71 ± 0.13 ^bcd^	37.22 ± 1.15 ^fg^
3	0.062	45	6	1.5	3.16 ± 0.07 ^ghij^	33.10 ± 1.33 ^hijkl^
4	0.069	45	4	1.0	3.57 ± 0.29 ^cde^	36.88 ± 1.64 ^fgh^
5	0.069	50	5	2.0	2.89 ± 0.01 ^jkl^	43.37 ± 2.11 ^bc^
6	0.069	45	5	1.5	3.97 ± 0.08 ^ab^	46.34 ± 3.08 ^ab^
7	0.069	50	5	1.5	2.84 ± 0.12 ^lm^	36.52 ± 1.54 ^fgh^
8	0.062	45	4	1.5	3.12 ± 0.07 ^hijk^	41.85 ± 0.98 ^cde^
9	0.069	45	4	2.0	3.01 ± 0.09 ^ijkl^	36.38 ± 1.65 ^fgh^
10	0.069	40	5	1.0	3.28 ± 0.38 ^fghi^	30.72 ± 0.18 ^jkl^
11	0.062	45	5	2.0	2.92 ± 0.02 ^jkl^	31.46 ± 2.92 ^ijkl^
12	0.069	50	4	1.5	2.96 ± 0.10 ^jkl^	33.01 ± 2.53 ^ijkl^
13	0.062	45	5	1.0	2.60 ± 0.15 ^m^	39.21 ± 1.69 ^def^
14	0.069	45	6	2.0	3.37 ± 0.03 ^efgh^	34.99 ± 3.48 ^ghi^
15	0.069	45	5	1.5	4.10 ± 0.19 ^a^	39.82 ± 3.78 ^cdef^
16	0.077	45	4	1.5	3.61 ± 0.09 ^cde^	29.48 ± 2.26 ^lm^
17	0.077	45	5	1.0	3.43 ± 0.05 ^efg^	26.59 ± 2.86 ^m^
18	0.069	45	5	1.5	3.74 ± 0.16 ^bcd^	42.89 ± 2.55 ^bcd^
19	0.069	50	6	1.5	2.79 ± 0.05 ^lm^	38.87 ± 2.34 ^ef^
20	0.062	40	5	1.5	3.26 ± 0.07 ^fghi^	33.69 ± 0.97 ^ghijk^
21	0.069	50	5	1.0	2.60 ± 0.07 ^m^	37.58 ± 1.76 ^fg^
22	0.069	40	6	1.5	3.48 ± 0.12 ^def^	30.54 ± 1.45 ^jkl^
23	0.077	45	5	2.0	2.86 ± 0.06 ^klm^	40.04 ± 2.78 ^cdef^
24	0.069	40	4	1.5	3.35 ± 0.04 ^efgh^	34.07 ± 1.38 ^ghij^
25	0.069	45	5	1.5	3.76 ± 0.14 ^bc^	43.02 ± 0.67 ^bcd^
26	0.069	45	5	1.5	3.58 ± 0.40 ^cde^	48.55 ± 1.84 ^a^
27	0.069	40	5	2.0	3.36 ± 0.03 ^efgh^	34.41 ± 0.74 ^ghij^
28	0.077	40	5	1.5	3.14 ± 0.15 ^hij^	30.12 ± 2.49 ^klm^
29	0.062	50	5	1.5	2.20 ± 0.08 ^n^	40.07 ± 1.38 ^cdef^

Notes: Different lowercase letters within the same column indicate statistically significant differences among samples for tensile strength and elongation at break, respectively (*p* < 0.05).

**Table 3:** ANOVA of regression model with tensile strength as the response variable.

Source of Variation	Sum of Squares (SS)	Degrees of Freedom (df)	Mean Square (MS)	F-Value	*p*-Value	Significance
Model	4.88	14	0.3484	11.31	<0.0001	**
A—Starch Content	0.4524	1	0.4524	14.68	0.0018	**
B—Plasticizer Content	1.07	1	1.07	34.85	<0.0001	**
C—WPI Content	0.0012	1	0.0012	0.0389	0.8464	
D—GA Concentration	0.0075	1	0.0075	0.2434	0.6294	
AB	0.1444	1	0.1444	4.69	0.0482	*
AC	0.0009	1	0.0009	0.0292	0.8668	
AD	0.1980	1	0.1980	6.43	0.0238	*
BC	0.0225	1	0.0225	0.7301	0.4072	
BD	0.0110	1	0.0110	0.3578	0.5593	
CD	0.1225	1	0.1225	3.98	0.0660	
A^2^	1.16	1	1.16	37.65	<0.0001	**
B^2^	1.69	1	1.69	54.84	<0.0001	**
C^2^	0.0773	1	0.0773	2.51	0.1356	
D^2^	0.9824	1	0.9824	31.88	<0.0001	**
Residual	0.4314	14	0.0308			
Lack of Fit	0.2634	10	0.0263	0.6272	0.7499	
Pure Error	0.1680	4	0.0420			
Total Variation	5.31	28				

Notes: * indicates statistical significance (*p* < 0.05), ** indicates high statistical significance (*p* < 0.01).

**Table 4:** ANOVA of regression model with elongation at break as the response variable.

Source of Variation	Sum of Squares (SS)	Degrees of Freedom (df)	Mean Square (MS)	F-Value	*p*-Value	Significance
Model	721.29	14	51.52	8.38	0.0001	**
A—Starch Content	31.40	1	31.40	5.11	0.0403	*
B—Plasticizer Content	107.22	1	107.22	17.45	0.0009	**
C—WPI Content	2.81	1	2.81	0.4577	0.5097	
D-GA Concentration	28.61	1	28.61	4.66	0.0488	*
AB	0.0001	1	0.0001	0.0000	0.9968	
AC	67.98	1	67.98	11.06	0.0050	**
AD	112.36	1	112.36	18.28	0.0008	**
BC	22.04	1	22.04	3.59	0.0791	
BD	1.10	1	1.10	0.1794	0.6783	
CD	4.73	1	4.73	0.7697	0.3951	
A^2^	143.15	1	143.15	23.29	0.0003	**
B^2^	116.91	1	116.91	19.02	0.0007	**
C^2^	157.36	1	157.36	25.60	0.0002	**
D^2^	118.29	1	118.29	19.25	0.0006	**
Residual	86.05	14	6.15			
Lack of Fit	40.28	10	4.03	0.3521	0.9177	
Pure Error	45.77	4	11.44			
Total Variation	807.33	28				

Notes: * indicates statistical significance (*p* < 0.05), ** indicates high statistical significance (*p* < 0.01).

**Table 5:** Changes in starch short-range order during the experiment.

Experimental Process	Short-Range Order (DO) of Starch (1047 cm^−1^/1022 cm^−1^)
Native Starch	0.90 ± 0.00 ^h^
Add WPI	0.97 ± 0.00 ^c^
50 °C	0.97 ± 0.00 ^c^
60 °C	0.97 ± 0.00 ^b^
70 °C	0.97 ± 0.00 ^a^
80 °C	0.97 ± 0.00 ^d^
90 °C	0.95 ± 0.00 ^e^
95 °C	0.94 ± 0.00 ^g^
Add GA	0.95 ± 0.00 ^f^
Experiment completed	0.95 ± 0.01 ^f^

Notes: Different lowercase letters in the same column indicate significant differences *p* < 0.05. Standard deviations were rounded to two decimal places; therefore, values smaller than 0.005 are reported as 0.00.

**Table 6:** Water distribution in starch films during drying.

Drying Time (h)	Peak Time (ms)			Peak Area (%)		
	T_21_	T_22_	T_23_	A_21_	A_22_	A_23_
0	9.42 ± 0.34 ^a^	0.12 ± 0.21 ^k^	384.47 ± 2.43 ^c^	1.43 ± 0.00 ^f^	0.00 ± 0.00 ^i^	97.07 ± 2.13 ^ab^
1	2.20 ± 1.02 ^de^	16.93 ± 0.14 ^g^	332.19 ± 1.33 ^d^	0.25 ± 0.01 ^h^	1.90 ± 0.38 ^fg^	97.85 ± 0.03 ^a^
2	4.45 ± 0.53 ^c^	29.04 ± 0.41 ^d^	290.55 ± 0.86 ^e^	0.14 ± 0.01 ^h^	3.57 ± 0.76 ^e^	96.29 ± 0.81 ^b^
3	0.98 ± 0.10 ^ef^	13.16 ± 0.58 ^h^	506.21 ± 1.34 ^a^	1.14 ± 0.37 ^fg^	3.20 ± 0.14 ^e^	95.67 ± 0.36 ^bc^
4	1.20 ± 0.91 ^ef^	23.51 ± 0.44 ^e^	218.98 ± 0.93 ^f^	0.86 ± 0.01 ^g^	4.43 ± 0.15 ^d^	94.72 ± 0.14 ^c^
5	6.70 ± 0.48 ^b^	1.90 ± 0.36 ^j^	134.64 ± 0.64 ^i^	1.03 ± 0.40 ^fg^	2.24 ± 0.07 ^f^	96.74 ± 0.05 ^ab^
6	1.14 ± 0.49 ^ef^	11.57 ± 0.67 ^i^	219.07 ± 0.81 ^f^	3.13 ± 0.45 ^e^	94.76 ± 0.56 ^a^	2.11 ± 0.61 ^e^
7	1.89 ± 0.86 ^ef^	33.93 ± 0.32 ^c^	409.23 ± 1.46 ^b^	6.13 ± 0.37 ^d^	91.01 ± 0.24 ^b^	2.86 ± 0.28 ^de^
8	3.21 ± 0.35 ^d^	36.15 ± 0.04 ^b^	384.62 ± 2.64 ^c^	7.01 ± 0.11 ^c^	89.15 ± 0.44 ^c^	3.84 ± 0.55 ^d^
9	1.63 ± 0.72 ^ef^	18.64 ± 0.85 ^f^	110.07 ± 0.53 ^j^	95.32 ± 0.57 ^b^	1.64 ± 0.63 ^fgh^	3.04 ± 0.06 ^de^
10	1.54 ± 0.70 ^ef^	50.53 ± 0.82 ^a^	145.49 ± 0.96 ^h^	96.35 ± 0.23 ^a^	1.12 ± 0.97 ^h^	2.54 ± 0.33 ^de^
11	0.97 ± 0.39 ^ef^	32.94 ± 1.07 ^c^	192.10 ± 1.33 ^g^	94.93 ± 0.14 ^b^	1.39 ± 0.53 ^gh^	3.69 ± 0.09 ^d^
12	0.57 ± 0.12 ^f^	15.95 ± 0.34 ^g^	103.35 ± 1.42 ^k^	95.11 ± 0.20 ^b^	1.30 ± 0.63 ^gh^	3.59 ± 0.26 ^de^

Notes: Different lowercase letters in the same column indicate significant differences (*p* < 0.05) according to Duncan’s multiple range test at a 95% confidence level, which indirectly reflects non-overlapping 95% confidence intervals among treatments.

## Data Availability

The original contributions presented in the study are included in the article, further inquiries can be directed to the corresponding author.
